# Sleep disturbances and depressive and anxiety symptoms during pregnancy: associations with delivery and newborn health

**DOI:** 10.1007/s00404-022-06560-x

**Published:** 2022-04-24

**Authors:** Hilla Peltonen, E. Juulia Paavonen, Outi Saarenpää-Heikkilä, Tero Vahlberg, Tiina Paunio, Päivi Polo-Kantola

**Affiliations:** 1 Department of Obstetrics and Gynecology, Turku University Hospital and University of Turku, Turku, Finland; 2 Pediatric Research Center, Child Psychiatry, University of Helsinki and Helsinki University Hospital, Helsinki, Finland; 3 Public Health Solutions, Finnish Institute for Health and Welfare, Helsinki, Finland; 4 Centre for Child Health Research, Tampere University and Tampere University Hospital, Tampere, Finland; 5 Department of Clinical Medicine, Biostatistics, University of Turku, Turku, Finland; 6 Department of Psychiatry, Helsinki University Hospital and University of Helsinki, Helsinki, Finland; 7 Finnish Institute for Health and Welfare, Helsinki, Finland; 8 Sleep Research Unit, University of Turku, Turku, Finland

**Keywords:** Sleep disturbances, Depression, Anxiety, Mother, Delivery, Newborn

## Abstract

**Background:**

Sleep disturbances and mood symptoms are common in late pregnancy; according to the literature, they can affect delivery and newborn outcomes. This study evaluated the effect of sleep and mood symptoms on delivery and newborn health, because there are insufficient and partly contradictory studies on the topic.

**Methods:**

A cohort of 1414 mothers in their third trimester was enrolled in this prospective cross-sectional questionnaire study. Validated questionnaires were assessed for the measurement of sleep disturbances and depressive and anxiety symptoms. The data on delivery and newborn outcomes were obtained from hospital medical records.

**Results:**

Sleep disturbances were very common. A higher insomnia score (*β* = − 0.06, *p* = 0.047) and longer sleep need (*β* = 0.07, *p* = 0.047) were related to delivery at a lower gestational age. In addition, a higher insomnia score (*β* = − 28.30, *p* = 0.010) and lower general sleep quality (*β* = − 62.15, *p* = 0.025) were associated with lower birth weight, but longer sleep duration and longer sleep need with a higher birth weight (*β* = 28.06, *p* = 0.019; *β* = 27.61, *p* = 0.028, respectively). However, the findings regarding birth weight lost their significance when the birth weight was standardized by gestational weeks. Concerning Apgar scores and umbilical artery pH, no associations were found. Snoring was associated with a shorter duration of the first phase of delivery (*β* = − 78.71, *p* = 0.015) and total duration of delivery (*β* = − 79.85, *p* = 0.016). Mothers with higher insomnia, depressive, or anxiety symptoms were more often treated with oxytocin (OR 1.54 95% CI 1.00–2.38, *p* = 0.049, OR 1.76, 95% CI 1.02–3.04, *p* = 0.049 and OR 1.91, CI 95% 1.28–2.84, *p* < 0.001, respectively) and those with higher depressive and anxiety symptoms were delivered more often with elective cesarean section (OR 4.67, 95% CI 2.04–12.68, *p* < 0.001 and OR 2.22, 95% CI 1.03–4.79, *p* = 0.042).

**Conclusions:**

Maternal sleep disturbances and mood symptoms during pregnancy are associated with delivery and newborn health. However, nearly, all the outcomes fell within a normal range, implying that the actual risks are low.

## Introduction

Pregnant mothers sleep poorly [1]. Sleep disturbances, especially insomnia symptoms, are common during pregnancy and often worsen toward the end of pregnancy [2–5]. Insomnia symptoms, including difficulties falling asleep (initiation insomnia), nightly awakenings, and too early morning awakenings (maintenance insomnia) can all lead to decreased sleep time and poor overall sleep quality [6]. The occurrence of sleep-disordered breathing (snoring, sleep apnea, and partial upper airway obstruction) also increases during pregnancy [5, 7], probably because of increased weight, edema, and nasal congestion [8].

Maternal sleep disturbances, especially poor sleep quality, short sleep duration, and sleep-disordered breathing, may contribute to maternal morbidity and adverse delivery outcomes, such as preterm delivery [9, 10]. In a study of 166 mothers by Okun et al. [11] poor sleep quality, especially in early pregnancy (14–16 weeks), but also with a tendency in late pregnancy, was a predictor of preterm birth. Disrupted sleep leading to stress system activation, inflammatory response rise, and changes in the hypothalamic–pituitary–adrenal axis (HPA) are suggested to be the potential underlying mechanisms [12]. Different ways to ease stress have been shown to improve pregnancy-related sleep disturbances, stress, anxiety, and depressive symptoms [13].

Previous studies have indicated that poor maternal sleep could also be associated with a longer duration of delivery [14–16] and may increase the risk of operative deliveries, especially cesarean section [14, 15, 17]. In an Iranian study of 457 primiparas [14], short sleep duration in late pregnancy was associated with a longer duration of all phases of delivery, and mothers sleeping longer were more likely to deliver vaginally. Another Iranian study of 88 mothers found that mothers sleeping less (6.45 ± 2.07 h vs. 8.47 ± 1.86 h) per night had longer deliveries and a 20% higher risk for cesarean delivery [18]. In addition, maternal sleep disturbances may increase the risks for the growing fetus; short sleep duration [14, 19] and habitual snoring [8, 20] have been shown to be associated with adverse newborn outcomes, such as lower birth weight and lower Apgar scores. However, the literature is not unanimous, with some studies not supporting the association between maternal sleep and delivery or neonatal outcomes [21–23]. The lack of consensus may be because of varying study designs and differences in clinical practice or inadequate control of confounding factors [21].

Low sleep quality and short sleep duration during pregnancy and the postpartum period are known risk factors for depressive and anxiety symptoms and vice versa [24–27]. Mood symptoms during pregnancy and the postpartum period have been linked and are often screened for but still underdiagnosed [28]. The most common symptoms during pregnancy—especially postpartum—are anxiety in its variations, excessive worry for fetal or newborn health, cognitive-affective depressive symptoms, suicidal or self-judgmental thoughts, and somatic symptoms such as sleep, fatigue, and appetite changes [29]. In a large systematic review article by Grigoriadis et al. [30] that included 29 original articles, anxiety during pregnancy was associated with adverse newborn outcomes such as preterm delivery, as well as lower birth weight and a smaller head circumference. However, maternal anxiety and newborn Apgar points were not associated. In a comprehensive review article written by the same author [31], maternal depression during pregnancy was associated with increased odds of preterm delivery and decreased breastfeeding initiation but not with other delivery or newborn outcomes. Depressive and anxiety symptoms have also been linked with a higher occurrence of cesarean section [32], but contradictory findings have also been presented [21, 30, 33].

The aim of our study was to evaluate the associations between maternal sleep disturbances and mood symptoms and delivery and newborn health. We concentrated on insomnia symptoms and sleep duration, as well as depressive and anxiety symptoms. We hypothesized that both sleep disturbances and mood symptoms would increase the risk of delivering earlier, having a longer delivery, and having an operative delivery while being related to poorer health in the newborn.

## Materials and Method

### Subjects

The current prospective cross-sectional study was a part of the larger Finnish CHILD-SLEEP birth cohort, which has been described in detail by Paavonen et al. [34]. Mothers were recruited by midwives or nurses working in maternity clinics in the Pirkanmaa area during routine pregnancy-related check-ups in late pregnancy (around gestational week 32). The researchers carefully trained the midwives and nurses regarding enrollment. All participants were given both oral and written information about the study. The mothers were eligible if they were willing to participate, had sufficient language skills (Finnish) to complete the study questionnaires, and if they gave their written consent to take part. To obtain a comprehensive and random population for the study, mothers with basic diseases were also included. Altogether, 1,673 mothers participated in the study, of which 315 (19.3%) had one and 37 (2.3%) had more than one somatic disease. Of all, nine mothers (0.6%) had a disability. A total of 629 (42%) had a somatic disease such as asthma, allergy, diabetes, hypertension, epilepsy, or sleep apnea, and 58 (3.9%) had a psychiatric disease such as ADHD, depression, anxiety or panic disorder, or schizophrenia.

**Fig. 1:**
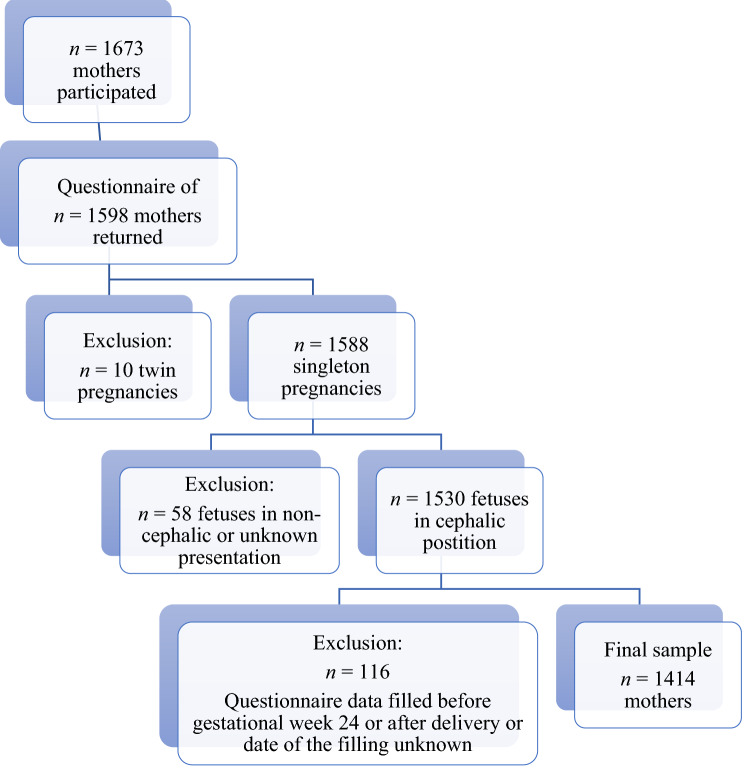
Flowchart of the recruitment process

Altogether, 1,598 questionnaires were returned. Mothers who had an incomplete questionnaire, missing delivery or newborn data, or completed the questionnaire before gestational week 24 or after the delivery were excluded (*n* = 116). The mothers filled in the questionnaire on average during gestational week 35 (range 24–41). Because the delivery outcomes were of special interest in our study, we also excluded twin pregnancies (*n* = 10) and pregnancies with a fetus other than a cephalic presentation (*n* = 58). A final sample of 1,414 mothers remained. The mothers were recruited between April 2011 and December 2012, and the infants were born between April 2011 and February 2013. During that time period, approximately 7,700 infants fulfilling the inclusion criteria were born in the target area, but because of the exclusion criteria, maternal refusals, language difficulties, and a failure on the prenatal nurses’ part to present the survey to the mothers, the sample coverage was approximately 20% (Fig. 1).

### Questionnaires

The sleep disturbances were evaluated using 11 questions drawn from the Basic Nordic Sleep Questionnaire (BNSQ) [35] (general sleep quality, difficulties falling asleep, nightly awakenings per week, number of awakenings per night, too early awakenings, self-reported snoring, sleep duration, and sleep need). General sleep quality was rated on a 5-point scale as 1 = ‘good’, 2 = ‘quite good’, 3 = ‘intermediate (neither good nor poor)’, 4 = ‘quite poor’ or 5 = ‘poor’, the frequency of nocturnal awakenings per night 1 = ‘none’, 2 = ‘once’, 3 = ‘twice’, 4 = ‘three to four times’, 5 = ‘at least five times’, and the other questions with a 5-point scale as 1 = ‘never or less than once a month’, 2 = ‘less than in 1 day a week’, 3 = ‘in 1 or 2 days a week’, 4 = ‘in 3–5 days a week’, and 5 = ‘daily or almost daily’ (Appendix).

To represent the severity of cooperative action of all the insomnia symptoms (general sleep quality, difficulties to fall asleep per week, nightly awakenings per week, number of awakenings per night, too early awakenings per week), the responses of all five questions were dichotomized (0 = ‘1–2 times per week/night or less [responses 1–3]’ vs. 1 = ‘3–5 times or more per week/night’ [responses 4–5]), and the scores were summed to form an insomnia score (range 0–5 points). A sum score of 4 points or more was considered deviant, because then, the mother would have had at least four different sleep disturbances occurring at least 3–5 times per week/night. Sleep duration (hours, h) was calculated as the average self-reported sleep time during weekdays and weekends and sleep need as the self-reported desired sleep time. If the sleep times ranged over 2 h, the reply was excluded. Sleep loss was defined by subtracting sleep needs from sleep time. Sleep duration < 6 h and sleep loss > 2 h were defined as deviant.

Depression was evaluated using the shortened 10-item version of the Center for Epidemiologic Studies Depression Scale (CES-D) [36] with 10 questions on a scale from 0 to 3 for each question. The scores were totaled to form a depression score (range 0–30) and used in analyses both as a continuous and categorical (a total score ≥ 12 points [95th percentile] was used as the cut-off point). A shortened State-Trait Anxiety Inventory (STAI) [37] was used to evaluate anxiety, with six questions on a scale from 1 to 4 for each question (anxiety at all times and the person’s vulnerability to anxiety). The scores were totaled to form an anxiety score (range 6–24) and used in analyses both as a continuous and categorical (a total score ≥ 12 [95th percentile] was used as the cut-off point) (Appendix).

### Data on delivery and newborn health

Delivery and newborn health data were collected from hospital medical records and hospital register data. Maternal delivery variables included gestational weeks at the time of delivery, duration of delivery (stage I [min], stage II [min], and total duration [stage I + stage II, min]), the type of delivery (spontaneous vaginal/vacuum delivery/elective cesarean section/acute cesarean section), and the use of oxytocin for induction or augmentation during delivery (yes/no). The start of stage I was determined by one contraction lasting for at least one minute and the contractions lasting continuously for at least 2 h with an interval of 5 min or less. Stage I lasted until stage II began. The start of stage II was determined by a fully dilated cervix and the need for pushing. Stage II lasted until the birth of the newborn. In some cases, the delivery was already ongoing when the mother entered the hospital, so the onset of the delivery was retrospectively determined by the anamnesis given by the mother. The newborn variables were weight (grams), standardized birth weight (= *z* score [a standard deviation score], which was calculated as the number of standard deviations an individual birth weight differs from population-based mean birth weight value at a certain gestational week of pregnancy), Apgar scores (at 1 min and 5 min), and pH of the umbilical artery (uApH) and umbilical vein (uVpH) at birth.

### Statistical analyses

Sleep variables, depressive and anxiety symptoms, delivery and newborn variables, and basic characteristics were first submitted for descriptive analysis and were expressed as means and standard deviations (SD) and ranges or frequencies (numbers and percentages). Insomnia total score, sleep duration, sleep loss, CES-D total score (depression score), and STAI total score (anxiety score) were used both as continuous and dichotomous variables (cut-off points: Insomnia score ≥ 4, sleep duration < 6 h, sleep loss > 2 h, CES-D ≥ 12, STAI ≥ 12). Sleep need was considered continuous and snoring considered dichotomized (no = ‘1–2 times per week/night or less’ vs. yes = ‘3–5 days or more a week’). Maternal age and BMI were considered continuous. Delivery variables were considered categorized, save for gestational weeks at birth, which were calculated as continuous. Newborn variables were considered as continuous, except for the Apgar scores, which were categorized as ≤ 7 or > 7 (both at 1 min and 5 min).

Finally, we conducted a series of regression models to control for potentially confounding background factors (age, parity, BMI, general health, smoking, and education). Linear regression models were used to study those factors related to gestational age, birth weight and standardized birth weight, duration of delivery (stages I and II and total duration), and birth variables (Apgar scores at 1 and 5 min and uApH). Logistic regression was used to study the odds of oxytocin use and elective cesarean section. The cases with cesarean section were excluded from those models, where birth variables or duration of delivery were studied (the *n* in the models varied between 1258 and 1268).

In the modeling, each explanatory factor was studied separately to control for confounding factors in the statistical models. *p* values of < 0.05 were considered statistically significant and are bolded in the tables. The power calculations show that with sample size about 1400 (1429) we can detect OR’s of about 1.3 when the alpha level (level of significance) is set at 0.05 and the power is set at 0.8. Statistical computations were performed using the SPSS Statistics 26 data program.

## Results

### Basic characteristics

Maternal characteristics are shown in Table 1. Sociodemographic factors included age (years), parity (nulliparous/multiparous), and education (low [no education or vocational training]/intermediate/high [university]). Health behavior factors included body mass index at the time of the survey (BMI, kg/m^2^) and smoking (yes/no).

**Table 1:** Basic characteristics

	*n*	Mean (SD)	%	Range
Age (years)	1411	30.6 (4.6)	17–48	17–48
BMI (kg/m^2^)	1376	28.4 (4.4)	19.2–47.6	19.2–47.6
Vocational education	1382			
None or some vocational training	99		7.2%	
Vocational degree or polytechnic	797		57.7%	
University	486		35.2%	
Parity	1319			
Nulliparous	612		46.4%	
Smoking during pregnancy	1409			
Yes	84		6.0%	

*BMI* body mass index

### Maternal sleep quality and mood symptoms

Sleep disturbances, sleep durations, and depressive and anxiety symptoms are described in Tables 2, 3. The most common sleep disturbance was nocturnal awakenings, with 98.6% of the mothers experiencing these weekly and 83.4% daily.

**Table 2:** Maternal sleep duration, insomnia and mood symptoms

	*n*	Mean (SD)	%	Range
Sleep duration (min)	1408	484 (63)		180–720
Sleep duration < 6 h	59		4.2	
Sleep need (min)	1403	529 (60)		
Sleep loss (min)	1398	45 (63)		300–1140
Sleep loss > 2 h	100		7.2	− 240 to + 600
Insomnia score	1406	1.8 (1.1)		0–5.0
Depression score (CES-D)	1410	5.2 (3.5)		0–23.0
Anxiety score (STAI)	1413	9.0 (2.4)		6–21.0

*CES-D* Center for Epidemiologic Studies Depression Scale, *STAI* State-Trait Anxiety Inventory

**Table 3:** Maternal sleep quality and specific sleep disturbances

	Total *n*	*n* (%)	*n* (%)	*n* (%)	*n* (%)	*n* (%)
		Never or less than once a month or night	Less than one day a week or night	On 1–2 days a week or night	On 3–5 days a week or night	Daily or almost daily
Difficulties to fall asleep	1414	495 (35.0)	428 (30.3)	295 (20.9)	133 (9.4)	63 (4.5)
Awakenings per week	1413	4 (0.4)	14 (1.0)	57 (4.0)	160 (11.3)	1178 (83.4)
Awakenings per night	1408	23 (1.6)	394 (28.0)	490 (34.8)	436 (31.0)	65 (4.6)
Too early awakenings	1413	507 (35.9)	454 (32.1)	305 (21.6)	114 (8.1)	33 (2.3)
Snoring	1360	831 (61.1)	196 (14.4)	142 (10.4)	69 (5.1)	122 (9.0)

	Total *n*	*n* (%)	*n* (%)	*n* (%)	*n* (%)	*n* (%)
		Well	Quite well	Intermediate	Quite poor	Poor
General sleep quality	1414	194 (13.7)	550 (38.9)	284 (20.1)	330 (23.3)	56 (4.0)

### Delivery and newborn outcomes

Of all the mothers, 98.2% had a full term pregnancy (≥ 37 gestational weeks, range 33–42), and 82.3% delivered vaginally. Of the operative deliveries, the cesarean section rate was low—10.0%—and the vacuum extraction rate was 7.6%. Oxytocin (induction or augmentation) was used in over half of the deliveries (Table 4).

**Table 4:** Delivery and newborn outcomes

	*n*	Mean (SD)	%	Range
Gestational age at delivery (weeks)	1414	40.1 (1.2)		33.0–42.7
Delivery < 37 gestational weeks	25		1.8	
Delivery type	1410			
Spontaneous vaginal	1162		82.4	
Vacuum	106		7.5	
Elective caesarean	41		2.9	
Acute caesarean	101		7.2	
Duration of delivery				
Duration stage I (min)	1407	480 (350)		10–2315
Duration stage II (min)	1268	21 (19)		1–114
Total duration (min)	1407	511 (362)		10–2357
Oxytocin use	1411			
Yes	811		57.5	
Birth weight (gram)	1414	3597 (449)		1950–5780
Birth weight z-score	1414	− 0.1 (0.9)		− 2.8 to + 6.4
Birth length (cm)	1414	50.5 (1.9)		42.0–58.0
Birth length *z*-score	1414	0.0 (1.0)		− 3.9 to + 4.9
Head circumference (cm)	1412	35.0 (1.4)		30.5–40.0
Head circumference *z*-score	1412	0.0 (1.0)		− 3.0 to + 3.6
Apgar scores				
1 min	1403	8.5		1–10
5 min	1401	8.9		3–10
Newborn pH				
Artery	1398	7.3		6.8–7.6
Vein	203	7.3		7.0–7.5

Of all the newborns, 1.2% (*n* = 19) had a standardized birth weight under − 2 SD and 2% (*n* = 28) over + 2 SD. Umbilical artery pH (uApH) was normal for most of the newborns: 3.2% (*n* = 45) had a uApH < 7.10 and only 0.1% (*n* = 2) had a uApH < 7.00. The vein pH was available only in 203 newborns, so it was not included in our analysis. The 1-min Apgar scores were < 7 in 3.1% (*n* = 44), and the 5-min Apgar scores were < 7 in 0.1% (*n* = 4). The newborn data are shown in Table 4.

### Associations between maternal sleep quality and mood symptoms and delivery outcomes

Mothers with a higher insomnia score delivered at a lower gestational age (Table 5); a 1-point increase in the insomnia score shortened the duration of pregnancy, on average, by a half (0.5) day (0.06 week). In addition, longer sleep need was associated with a slightly (0.5 days) longer duration of pregnancy (Table 5). The results remained after controlling for other sleep variables and mood symptoms. However, the mean gestational week at delivery fell within the normal range in the entire sample. Sleep loss was associated with a longer duration of phase 1 and longer total duration of delivery (Table 6). Instead, snoring was associated with a shorter duration of phase I and a shorter total duration of delivery.

**Table 5:** Associations between maternal sleep quality, mood symptoms and gestational age and birth weight in all mothers

Predictor variable	Gestational age (weeks)^b^		Birth weight (grams)^b^		Birth weight (*z*-score)^b^	
	Adjusted^a^ *β* (SE)	*p* value	Adjusted^a^ *β* (SE)	*p* value	Adjusted^a^ *β* (SE)	*p* value
Sleep duration (h)	0.05 (0.03)	0.137	**28.06 (12.00)**	**0.019**	0.05 (0.02)	0.053
Sleep need (h)	**0.07 (0.04)**	**0.047**	**27.61 (12.53)**	**0.028**	0.04 (0.03)	0.126
Sleep loss (h)	0.01 (0.03)	0.767	− 2.70 (11.76)	0.818	− 0.01 (0.02)	0.656
Insomnia score	− **0.06 (0.30)**	**0.047**	− **28.30 (10.94)**	**0.010**	− 0.04 (0.02)	0.065
Sleep quality	− 0.11 (0.08)	0.176	− **62.15 (27.70)**	**0.0025**	− 0.10 (0.06)	0.076
Snoring	0.10 (0.12)	0.391	71.79 (43.26)	0.097	0.13 (0.09)	0.149
Depression score (CES-D)	− 0.01 (0.01)	0.565	− 0.69 (3.59)	0.847	0.00 (0.01)	0.784
Anxiety score (STAI)	− 0.01 (0.01)	0.627	− 4.09 (5.22)	0.434	− 0.01 (0.01)	0.524

*β* adjusted regression coefficient, *SE* standard error, *CES-D* Center for Epidemiologic Studies Depression Scale, *STAI* State-Trait Anxiety inventory, *z-score* (a standard deviation score) calculated as a number of standard deviations an individual birth weight differs from population-based mean birth weight value at a certain gestational week of pregnancy

^a^All models are adjusted for age, parity, BMI, general health, smoking and education. All variables are considered as continuous variables except for snoring which was considered as categorical (yes vs no)

^b^All mothers *n* = 1410

Bolded values are the values which are significant (*p*<0.05)

**Table 6:** Associations between maternal sleep quality and mood symptoms and duration of delivery in mothers with vaginal (spontaneous or assisted) delivery

Explanatory variable	I stage^b^		II stage^b^		Total duration (I + II)^b^	
	Adjusted^a^ *β* (SE)	*p* value	Adjusted^a^ *β* (SE)	*p* value	Adjusted^a^ *β* (SE)	*p* value
Sleep duration (h)	− 0.011 (0.01)	0.124	0.011 (0.012)	0.360	− 0.010 (0.007)	0.151
Sleep need (h)	0.010 (0.01)	0.213	0.004 (0.012)	0.749	0.010 (0.007)	0.199
Sleep loss (h)	**0.019 (0.01)**	**0.001**	− 0.007 (0.001)	0.525	**0.017 (0.007)**	**0.012**
Insomnia score	-0.001 (0.07)	0.905	0.003 (0.01)	0.759	0.00 (0.006)	0.959
Snoring	− **0.07 (0.03)**	**0.010**	− 0.057 (0,043)	0.181	− **0.063 (0.026)**	**0.015**
Depression score (CES-D)	0.001 (0.002)	0.815	0.001 (0.003)	0.865	0.00 (0.002)	0.823
Anxiety score (STAI)	− 0.001 (0.003)	0.778	0.004 (0.005)	0.433	− 0.001 (0.003)	0.787

*β* adjusted regression coefficient, *SE* standard error, *CES-D* Center for Epidemiologic Studies Depression Scale, *STAI* State-Trait Anxiety inventory

^a^All models are adjusted for age, parity, BMI, general health, smoking and education

^b^Models are performed only in mothers with vaginal delivery (spontaneous vacuum assisted, *n* = 1268). All outcome variables were log transformed before analyses. All variables are considered as continuous variables except for snoring which was considered as categorical (yes vs no)

Bolded values are the values which are significant (*p*<0.05)

When considered as categorical, a high level of insomnia score, a high level of depression score, and a high-level anxiety score were related to higher odds for being treated with oxytocin during delivery and higher depressive and anxiety scores with higher odds for elective (but not with acute) cesarean section (Table 7). No other associations with delivery outcomes were found.

**Table 7:** Odds rations for oxytocin use in women with vaginal delivery and risk for elective cesarean section in all women

Explanatory variable	Oxytocin use^b^		Elective Caesarean section^c^	
	Adjusted^a^ OR (95% CI)	*p* value	Adjusted^a^ OR (95% Cl)	*p* value
Sleep duration		0.481		0.430
≥ 6 h	1.0		1.0	
< 6 h	1.27 (0.65–2.49)		1.66 (0.47–5.81)	
Sleep loss		0.553		0.497
≤ 2 h	1.0		1.0	
> 2 h	0.86 (0.53–1.41)		1.46 (0.49–4.33)	
Insomnia score		**0.049**		0.743
< 4	1.0		1.0	
≥ 4	**1.54 (1.00–2.38)**		0.84 (0.29–2.44)	
Snoring		0.969		0.074
No	1.0		1.0	
Yes	1.01 (0.64–1.60)		2.17 (0.03–4.40)	
Depression score		**0.044**		** < 0.001**
(CES-D) < 12	1.0		1.0	
(CES-D) ≥ 12	**1.76 (1.02–3.04)**		**4.67 (2.04–10.68)**	
Anxiety score		**0.001**		**0.042**
(STAI) < 12	1.0		1.0	
(STAI) ≥ 12	**1.91 (1.28–2.84)**		**2.22 (1.03–4.79)**	

*OR* adjusted odds ratio, *CI* confidence interval, *CES-D* Center for Epidemiologic Studies Depression Scale, *STAI* State-Trait Anxiety inventory

^a^All models are adjusted for age, parity, BMI, general health, smoking and education. All explanatory variables are considered as categorical variables

^b^Analysis for oxytocin usage was performed only in mothers with vaginal delivery (spontaneous or vacuum assisted, *n* = 1268)

^c^All mothers *n* = 1410

Bolded values are the values which are significant (*p*<0.05)

### Associations between maternal sleep quality and mood symptoms and newborn outcomes

Mothers with higher insomnia scores and lower general sleep quality delivered infants with lower weights (Table 5). Furthermore, those with longer sleep duration and longer sleep needs delivered infants with higher weight. However, when the gestational week at delivery was considered, here using standardized birth weight as the outcome, all these findings lost their statistical significance (Table 5). Concerning Apgar scores and uApH, no associations between the sleep variables or mood symptoms were found (data not shown).

All the above-mentioned results remained when the other sleep variables and mood symptoms were considered in the statistical modeling (data not shown).

## Discussion

In our sample, which comprised late gestational week pregnancies, both insomnia and sleepiness symptoms were very common. We found some specific correlations between sleep disturbances and mood symptoms and between delivery and newborn outcomes. However, the absolute risks were small, so their clinical significance remains unclear. Our sample was recruited relatively late in the third trimester, so the actual insomnia symptoms may have been present for only a short time frame, which could explain our weaker findings. Thus, more studies are warranted, particularly using follow-up samples starting from early on in the pregnancy.

Poor maternal sleep during pregnancy is a risk factor for preterm delivery [9, 10]. Although we could not confirm the finding of prematurity, we found that insomnia symptoms were associated with delivery in earlier gestational weeks, though the effect was low. This might be explained by the small number of preterm deliveries in our study population. We also found that longer sleep duration and higher sleep need were associated with a slightly longer duration of pregnancy. This finding supports the hypothesis that sufficient sleep leads to a better pregnancy outcome. Of note, however, was that sleep loss, as calculated by subtracting sleep need from sleep duration, was not associated with delivery weeks or newborn variables.

Prior research concerning maternal sleep disturbances and the duration of delivery is limited and partly controversial. Insomnia symptoms and short sleep duration, especially during the last trimester, have been suggested to predispose mothers to a longer duration of delivery [14, 18]. We found partly similar results; sleep loss was associated with a longer first stage and total time of delivery. On the other hand, in our study, neither sleep disturbances, sleep quality, nor total sleep duration were associated with the duration of delivery. This is consistent with an American study with 99 mothers [22] that found no effect of sleep quality or sleep duration on the duration of delivery stages. One explanation for the inconsistencies in the results could be the varying clinical practices between the countries and differences in the ways of recording the duration of the delivery.

Concerning the mode of delivery, in the group of 131 American mothers [15], sleeping less than 6 h per night one week before delivery was a risk factor for unplanned cesarean section. Moreover, the two earlier described Iranian studies [14, 18] found that both low sleep quality and short sleep duration in the third trimester were risk factors for cesarean section in general. In a large Swedish study [38], the researchers retrospectively screened the electronic perinatal records of 6,467 primiparas for free-text words that indicated stress, sleep disturbances, and worry, finding that the existence of these words in the charts predicted an increased risk for an emergency cesarean section. In addition, in a Taiwanese study of 120 mothers [39], poor sleepers in the third trimester were more likely to have a vacuum-assisted delivery. However, we could not confirm the associations between sleep disturbances and the mode of delivery. We found no correlation between maternal sleep and cesarean section, neither elective nor acute, which is in line with the results of the American study [22] and also with a Canadian study of 624 mothers [21]. Of note is that the assessment of sleep disturbances in previous studies has varied widely, and structured sleep questionnaires, as used in our study, have been seldom utilized. Furthermore, the frequencies of instrumental deliveries—especially the rates of cesarean section—range considerably between the studies (and countries) from our 10% to even up to 55% [17, 18, 21].

According to our results, snoring was associated with delivery duration; however, in contrast to our expectations, it was associated with a shorter delivery duration. The reason for this finding is unclear, and its meaning remains uncertain. Earlier, in a large American study of 1,673 mothers, snoring during pregnancy was associated not only with a lower birth weight, but also with a higher risk of elective and emergency cesarean section [8]. In another study [40], however, no association between snoring and delivery was found. Nevertheless, in our study, snoring did not relate to other delivery or newborn outcomes, so this finding could also be a random association. Comparing previous studies is challenging, because the methodology varies between the studies.

Depressive symptoms prior to delivery have been reported to increase the risk of emergency cesarean section [32]. We found that severe mood symptoms—both depressive and anxiety symptoms—were associated with elective cesarean section: mothers with a higher depressive score had almost five times and mothers with higher anxiety scores over two times higher incidence. No association with emergency cesareans emerged. Our finding of the risk of elective cesarean is probably explained by fear of childbirth. Mood symptoms, anxiety, and depression often coexist with the fear of child birth [41], and willingness to undergo a cesarean section among these mothers is common. Today, fear of giving birth is the leading cause for elective cesarean in Finland. The importance of our finding was notable, especially because the cesarean section rate in our study was low, because the sample was recruited in the third trimester and breech and twin pregnancies were excluded. The overall elective cesarean section rate in Finland was 7.0% in 2019 (thl.fi). Evidence suggests that mothers with perinatal anxiety and trauma-related disorders, as well as perinatal depression, have a higher risk for negative delivery or newborn outcomes [42].

High insomnia score, high depressive score, and high anxiety score correlated with the use of oxytocin during delivery. These findings were novel. Oxytocin causes contractions of the uterus during delivery and stimulates lactation [43]. It also plays an important role in increasing maternal–fetal trust and bonding while modulating fear, stress, and anxiety [44]. Anxiety and mood symptoms, that occur in the third trimester and during delivery have been shown to have negative effects on the duration of all phases of delivery [45]. In addition, in a recent large retrospective study, mothers exposed to additional oxytocin during delivery were at a higher risk for the development of postpartum depressive and anxiety disorders [46]. Mood symptoms often coexist with insomnia, so the finding of all these symptoms leading to the need for oxytocin is rational. It is possible that mothers suffering from insomnia or mood symptoms have lower levels of oxytocin during delivery, or they have a decreased binding ability of oxytocin to the uterine oxytocin receptors. This might lead to the need for an additional oxytocin stimulus. Unfortunately, in our cohort, we could not reliably find out whether the oxytocin used during deliveries was for induction or augmentation. In addition, the use of oxytocin during delivery also depends on the physician’s and midwife’s policy and can vary widely. Because oxytocin is important in maternal–fetal bonding and presumably is lower in mothers with anxiety or depressiveness, more research is needed to better understand the possible associations. Furthermore, treating mood symptoms in pregnant mothers with either medical or nonmedical treatment may reduce the risks for negative perinatal outcomes [42, 47].

There are only a few studies addressing the relationship between maternal sleep and mood symptoms and newborn outcomes, and most of these studies concentrate on maternal sleep duration. Sleep loss has been shown to negatively relate to fetal growth, leading to a lower birth weight [19]. We found that higher insomnia scores and lower general sleep quality were associated with lower birth weight, longer sleep duration, and longer sleep need with slightly higher birth weight. Nevertheless, when the birth weight was standardized with gestational age at delivery, all these associations disappeared. This emphasizes the importance of controlling for gestational length when studying birth weight. It has also been hypothesized that as a result of the suboptimal prenatal environment, the fetus has few resources at birth, resulting in lower Apgar scores [12]. According to an Iranian study with 457 participants, mothers sleeping less than 8 h per day in the third trimester have been shown to deliver newborns with lower Apgar scores compared with mothers sleeping longer [14]. Nonetheless, in that study, the clinical relevance of the findings remained unclear, because the Apgar scores of the newborns of short sleeping mothers were also within the normal range. In our study, no clinically relevant correlations emerged. This was also true in a Chinese study with 248 mothers and in a Canadian study with 650 mothers, where no correlations between maternal sleep variables and newborn health state at delivery were found [21, 39]. However, of note is that our study did not consider the effect in the case of very preterm newborns.

Our study comprised a large sample of pregnant Finnish mothers recruited during their third trimester and delivery and newborn data drawn from registers. Based on validation studies, the accountability and coverage of Finnish health care register data are high and dependable [48]. We used questionnaires, which have been shown to be valid and reliable [35] and have been used in similar studies before [49]. However, there were limitations. We recruited a random sample of mothers attending to their routine pregnancy-related check-ups and also included mothers with basic diseases. Thus, for instance, mothers with previous psychiatric comorbidities were enrolled, which could have interfered with our results regarding sleep and depressive and anxiety symptoms. However, the number of mothers with previously diagnosed psychiatric diseases was low—3.9%—and therefore, the bias can be considered marginal. In addition, by enrolling mothers with basic diseases, instead of healthy mothers only, our results could be better applied to the general population. Regarding the duration of the delivery, the duration of stage I could especially be partly biased, because in some mothers, the delivery was already ongoing when they entered the hospital; thus, the time of the onset was a retrospective estimation given by the mothers. In addition, variations in recordings done by the midwifes and physicians in charge were also possible. In our cohort, the cesarean section and vacuum-assisted delivery, as well as preterm delivery, rates were significantly lower than in the general population in Finland, so there might be selection bias in the results. Concerning cesarean section, the main reason for the low rate was the exclusion of breech presentation, twin pregnancies, and very preterm deliveries. The current study assessed maternal sleep and mood symptoms over the past few months before delivery and, therefore, can reliably present only the effect of sleep in late pregnancy. The current study was based on subjective questionnaires and no objective sleep data were collected. It is known that objective measurements of sleep can differ considerably from subjective self-reported sleep [50]. Nevertheless, the report errors were randomly distributed and, thus, equivalent for all participants. In addition, our cohort comprised of mothers delivering mainly full term, so our study did not consider the effects in the case of very preterm newborns, which means that the results cannot be interpreted in preterm cases.

## Conclusions

In our study, we found statistically significant associations between both sleep quality and mood symptoms and delivery and newborn outcomes, but the absolute risks were small. Although this finding can be considered favorable, sleep disturbances and mood symptoms are still major health issues during pregnancy. It is important to note that maternal sleeping problems and mood symptoms are clinically highly relevant regarding, for example, maternal subjective well-being and the risk for postpartum depression [49]. Therefore, these symptoms should also be considered possible risk factors for undesirable delivery outcomes and poorer newborn health. As healthcare professionals weigh the possible treatment or follow-up options for pregnant mothers, they should better take sleep and mood symptoms into consideration [42]. However, it might ease the burden of stress related to the course of pregnancy to know that the clinical risk related to insomnia and mood symptoms on delivery and newborn appear to be small. Finally, it is of note that our data represented only symptoms in late pregnancy, so our results cannot be extrapolated in the situation of mothers with long-term insomnia and mood symptoms. Therefore, future studies recruiting mothers in early pregnancy—or even before pregnancy—are needed.

## Data Availability

The data that support the findings are available from the Finnish Institute for Health and Welfare, but restrictions apply to the availability of these data, which were used under license for the current study, which means they are not publicly available. Data are, however, available from the authors upon reasonable request and with permission of the Finnish Institute for Health and Welfare.

## SLEEP (Questions drawn from Basic Nordic Sleep Questionnaire, BNSQ)

1. How often have you had difficulties falling asleep during the past month?
  - Never or less than once per month
  - Less than once per week
  - On 1—2 nights per week
  - On 3—5 nights per week
  - Every night or almost every night
2. How long time (on average, in minutes) do you stay awake in bed before you fall asleep (after lights off)?
  - During working days ________
  - During freetime ________
3. How often have you woken up at night during the past month?
  (f) Never or less than once per month
  (g) Less than once per week
  (h) On 1—2 nights per week
  (i) On 3—5 nights per week
  (j) Every night or almost every night
4. If you usually wake up during the night, how many times did you wake up during one night (during the past month)?
  - UsI do not wake up at night usually
  - Once per night
  - 2 times per night
  - 3–4 times per night
  - At least 5 times per night
5. How often have you awakened too early in the morning without being able to fall asleep again during the past month?
  - Never or less than once per month
  - Less than once per week
  - On 1—2 days per week
  - On 3—5 days per week
  - Daily or almost daily
6. How well have you been sleeping during the past month?
  - Well
  - Quite well
  - Neither well nor badly (intermediate)
  - Quite poorly
  - Poorly
7. How many hours do you usually sleep per night?
  - I usually sleep about _______hours per night
8. At what time do you usually go to bed (in order to go to sleep)?
  - During working week: at_________
  - During free days: at _________
9. At what time do you usually wake up?
  - During working week: at_________
  - During free days: at _________
10. Do you snore while sleeping? (ask other people if you are not sure)
  - never or less than once per month
  - less than once per week
  - on 1–2 days per week
  - on
  - 3–5 days per week
  - daily or almost daily
11. How many hours of sleep do you need per night (how many hours would you sleep if you had possibility to sleep as long as you need to)?
  - I need______ hours and _______min of sleep per night

Insomnia score, Scale 0–5.

To represent the severity of co-operative action of all the insomnia symptoms (general sleep quality, difficulties to fall asleep per week, nightly awakenings per week, number of awakenings per night, too early awakenings per week), the responses of all these five questions were dichotomized (0 = ’1–2 times per week/night or less [responses 1–3]’ vs 1 = ’3–5 times or more per week/night’[responses 4–5]) and a summary score was calculated by adding the dichotomized scores to form a insomnia score (range 0–5 points).

## Anxiety

Shortened and modified translated State-Trait Anxiety Inventory (STAI), 6 questions.

A person marks the most adequate answer for the feelings past the last month.

**Table:**

		Not at all	Somewhat	Moderately	Very much
1	I feel nervous and restless	O	O	O	O
2	I feel that difficulties are piling up so that I can’t overcome them	O	O	O	O
3	I worry too much over something that really doesn’t matter	O	O	O	O
4	I have disturbing thoughts	O	O	O	O
5	I take disappointments so keenly that I can’t put them out of my mind	O	O	O	O
6	I get in a state of tension or turmoil as I think over my recent concerns and interests	O	O	O	O

## Depressive symptoms

Center for epidemiologic Studies Depression Scale; 10 questions on a four-point scale.

A person marks the most adequate answer for the feelings past the last month.

**Table:**

		Not at all	A little	Some	A lot
1	I felt depressed	0	1	2	3
2	I felt that everything that I did was an effort	0	1	2	3
3	My sleep was restless	0	1	2	3
4	I was happy	3	2	1	0
5	I felt lonely	0	1	2	3
6	People were unfriendly	0	1	2	3
7	I enjoyed life	3	2	1	0
8	I felt sad	0	1	2	3
9	I felt that people dislike me	0	1	2	3
10	I could not “get going”	0	1	2	3

## Abbreviations

BNSQ: Basic Nordic Sleep Questionnaire
BMI: Body Mass Index
CES-D: Center for Epidemiologic Studies Depression Scale
STAI: State-Trait Anxiety Inventory
